# Supplementary material for: The Effects of Foods Embedded in Entertainment Media on Children’s Food Choices and Food Intake: A Systematic Review and Meta-Analyses
Source: Nutrients. 2020 Mar 31;12(4):964. doi: 10.3390/nu12040964 (PMC7230193; doi:10.3390/nu12040964)
Supplement: Supplementary file 1 [file nutrients-12-00964-s001.zip › Table S2_Search strategy.docx]

Effects of foods placed in entertainment media on children´s food choices and intake: a systematic review and meta-analyses

**SEARCH STRATEGY 🡪 18th July 2018**

**Academic Search Ultimate: 1794 records (788 academic journals)**

AB “product placement*” OR "food placement*" OR "food cue*" OR "brand placement" OR "placed product" OR "product placed" OR "food modeling" OR "adverg*"

**Business Source Ultimate: 2494 records (479 academic journals)**

AB “product placement*” OR "food placement*" OR "food cue*" OR "brand placement" OR "placed product" OR "product placed" OR "food modeling" OR "adverg*"

**PyscINFO: 951 records (839 academic publications)**

AB “product placement*” OR "food placement*" OR "food cue*" OR "brand placement" OR "placed product" OR "product placed" OR "food modeling" OR "adverg*"

**MEDLINE: 847 records (845 academic journals)**

AB “product placement*” OR "food placement*" OR "food cue*" OR "brand placement" OR "placed product" OR "product placed" OR "food modeling" OR "adverg*"

**PubMed: 302**

(“product placement*”[Title/Abstract] OR "food placement*"[Title/Abstract] OR "food cue*"[Title/Abstract] OR "brand placement"[Title/Abstract] OR "placed product"[Title/Abstract] OR "product placed"[Title/Abstract] OR "food modeling"[Title/Abstract] OR "adverg*"[Title/Abstract])
