# Supplementary material for: The Effects of Foods Embedded in Entertainment Media on Children’s Food Choices and Food Intake: A Systematic Review and Meta-Analyses
Source: Nutrients. 2020 Mar 31;12(4):964. doi: 10.3390/nu12040964 (PMC7230193; doi:10.3390/nu12040964)
Supplement: Supplementary file 1 [file nutrients-12-00964-s001.zip › Table S3. Summary of the studies.docx]

**Table S3.** Characteristics of studies included in the systematic review.

| Authors  (Year)  Journal | N | Mean age (SD) [range] % female | Placed target food(s) | Media | Manipulation | Behavioral outcomes | | Risk of bias | |
| --- | --- | --- | --- | --- | --- | --- | --- | --- | --- |
| Auty (2004) *Psychology & Marketing*  United Kingdom [59] | 105 | NR^1^  (NR)  [6-7]  54% [11-12]  46% | Pepsi (brand) | Movie  *(Home Alone)* | Experimental group was exposed to a scene of the movie with Pepsi placements.  Comparison group was exposed to another scene of the movie without Pepsi placements. | Choice:  Significant brand choice effects.  Greater effect when movie was previously seen. | | Low | |
| Brown  (2017)  *Appetite*  USA [60] | 114 | 9.82  (NR)  [9-11]  NR | Utz Cheese Balls (brand)  Lindor Chocolate Truffles (brand)  Banana  Snyder´s Pretzel Rods (brand) | Movie  *(Alvin and the Chipmunks)* | Experimental group was exposed to a movie that had 4 target foods placed. Target food placements were categorized by prominence according to the time the snack was featured.  Comparison group was exposed to another movie (Stuart Little) and no target food placements appeared. | Choice:  Significant effects of the most prominent snack (Utz Cheese Balls)  Intake  No significant effects on brand intake.  No significant effects when movie was previously seen. | | High | |
| Charry  (2014)  *International Journal of Advertising*  France[24] | 72 | 9.4  (1.17)  [8-11]  52.8% | Fruit  Fruit salad | TV show  *(Plus Belle la Vie)* | Experimental group was exposed to a scene of the TV show with bimodal food placements.  Comparison group was exposed to a scene of the TV show with unimodal food placements (only visual). | Choice:  Significant effects of bimodal placements on food choice. | Middle | |  |
| Dias  (2011)  *Journal of Consumer Behavior*  Portugal [44] | 231 | 7.47  (0.49)  [7-8]  49.4% | Potato chips  Cookies  Lollipop  Hamburger  Chocolate mousse  Banana  Milk  Strawberries  Fruit salad  Bread | Videogame^2^  *(designed for the experiment: grabbing foods)* | Experimental group 1 played a game where unhealthy snacks were placed.  Experimental group 2 played a game where healthy snacks were placed.  In both groups, participants get points when grabbing the foods. | Choice  Significant effects of type of game on food choice. | Middle | |  |
| Esmaeilpour  (2017)  *Journal of Food Products Marketing*  Iran [45] | 330 | 8.9  (NR)  [6-11]  69% | Potato chips  Biscuit  Sausage  Hamburger  Soda  Bread  Boiled egg  Milk  Pistachio | Videogame  *(designed for the experiment: grabbing foods)* | 2x2x2 factorial design between subjects design [Type of food (healthy vs. unhealthy), health knowledge (inactive vs. active) and entertainment experience (TV commercial vs. advergame)]. | Choice  Significant effects of type of food on food choice.  Greater effects for advergames (compared to advertising).  Greater effects when health knowledge was not activated. | Middle | |  |
| Folkvord  (2013)  *The American Journal of Clinical Nutrition*  Holland [46] | 270 | 8.9  (NR)  [8-10]  49.5% | Jelly cola bottle (brand)  Milk chocolate candy shell (brand)  Banana (brand)  Apple (brand) | Advergame  *(designed for the experiment: memory game)* | Experimental group 1 was exposed to a memory-game containing cards with unhealthy branded foods.  Experimental group 2 was exposed to a memory-game containing cards with healthy branded foods.  Comparison group 1 was exposed to a memory-game containing cards with toys.  Comparison group 2 was not exposed to the memory-game. | Intake  Significant effects of food advergames (both healthy and unhealthy memory-game) on unhealthy food intake  No significant differences between unhealthy and healthy food advergames on food intake. | Low | |  |
| Folkvord  (2014)  *Pediatrics*  Holland [47] | 261 | 7.7  (0.7)  [7-10]  48.8% | Jelly cola bottle (brand)  Milk chocolate candy shell (brand) | Advergame  *(designed for the experiment: memory game)* | 2x2x2 factorial design between subjects design [type of advergame (unhealthy vs. non-food), inhibition task (reward from refrain from eating vs. no reward) and impulsivity (low vs. high)]. | Intake  Significant effects of type of advergame on food intake.  No interaction effects neither of inhibition task nor impulsivity with type of advergame on food intake. | Middle | |  |
| Folkvord  (2015)  *Appetite*  Holland [48] | 92 | 8.42  (NR)  [7-10] | Candy (brand) | Advergame  *(designed for the experiment: brands placed as banners)* | 2x2 factorial design between subjects design [type of advergame (unhealthy vs. non-food) and attentional bias (high vs. low)]. | Intake  Significant effects of type of advergame on food intake.  No interaction effects between type of advergame and attentional bias. | Middle | |  |
| Folkvord  (2016)  *Preventive Medicine*  *Reports*  Holland [35] | 127 | 9.3  (1.5)  [8-11]  53% | Mandarin  Apple  Banana  Grapes | Videogame  *(designed for the experiment: memory game)* | Experimental group played a memory-game containing cards with fruits.  Comparison group played a memory-game containing cards with toys. | Intake  Significant effects on total fruit intake.  Significant effects for banana and mandarin, not for apple and grapes.  Sex, game attitude, hunger and BMI did not moderate the effects. | Middle | |  |
| Folkvord  (2016)  *Health Psychology*  NR [49] | 133 | 8.9  (1.0)  [7-10]  52.6% | Jelly cola bottle (brand)  Milk chocolate candy shell (brand) | Advergame  *(designed for the experiment: memory game)* | 2x2 factorial design between subjects design [type of advergame (unhealthy vs. non-food), go/no go task (no go food trials vs. no go control trials)]. | Intake  Significant effects of go/no go task (no go food trials) on unhealthy food intake.  Type of advergame did not influence the later intake (note: the intake was not while or immediately after the advergame). | Low | |  |
|  |  |  |  |  |  |  |  | |  |
| Folkvord  (2017)  *Appetite*  Holland and Spain [50] | 562 | 9  (1.43)  [6-12]  51.1% | Jelly cola bottle (brand) | Advergame  *(designed for the experiment: memory game)* | 2x2 factorial design between subjects design [type of advergame (unhealthy vs. non-food), protective message (present vs. absent)]. | Intake  Significant effects among Dutch children sample on unhealthy food intake (no effects among Spanish children).  No significant interaction between type of advergame and protective message. | Low | |  |
| Harris  (2012)  *Journal of Children and Media*  USA [40] | 152 | 9.4  (NR)  [7-12]  47.4% | Dole Foods fruits and vegetables (brand)  Oreo cookies (brand) and  Pop Tarts | Advergame  *(use a catapult to shoot foods into a barrel, encourage a maneuver snack man to eat healthy, dunk the cookies, catch falling Pop Tarts,)* | Experimental group 1 was exposed to a game containing unhealthy branded foods.  Experimental group 2 was exposed to a game containing healthy branded foods.  Comparison group was exposed to a game that did not contain foods (control group). | Intake  No significant effects (comparing with control group) either on healthy or unhealthy food intake.  Significant effects between unhealthy and healthy advergames conditions on unhealthy and healthy food intake respectively.  No significant age effects. | Middle | |  |
| Hudson  (2013)  *Journal of Food Products Marketing*  USA [51] | 225 | NR  (NR)  [7-12]  47.6% | Milk 2 go (brand)  Black diamonds Cheese rings (Brand)  Yoplait tubes (brand)  Dole fruit caps (brand)  Pepsi (brand)  Betty Crocker’s Fruit (brand)  Gushers (brand)  Reese’s Pieces (brand)  Frito-Lay Cheetos (brand) | TV show  *(Pop Idol)* | Experimental group 1 was exposed to a 20-min segment where 4 branded healthy foods were digitally inserted.  Experimental group 2 was exposed to a 20-min segment where 4 branded unhealthy foods were digitally inserted.  Comparison group was exposed to a 20-min segment without product placements. | Choice:  No significant effects of food placements on brand choice.  The majority of the children chose the same branded products.  No significant age differences. | Middle | |  |
| Mallinckrodt  (2007)  *Journal of Advertising*  Australia [52] | 295 | NR  (NR)  [5-8]  60% | Froot Loops cereal (brand) | Advergame  *(Throw a Froot Loop into the monster mouth and get more points and more mmm satisfaction than throwing pieces of fruits)* | Experimental group played 5-min game with a branded food placement before collecting data.  Comparison group did not play 5-min game with a branded food placement before collecting data. | Choice  Significant effects of food placement on brand and product category choice.  Greater effects for children aged 7-8 years.  Persuasive knowledge did not influence. | Middle | |  |
| Matthes  (2015)  *Journal of Consumer Behavior*  Austria [14] | 121 | 9.58  (2.49)  [6-14]  55% | Utz Cheese Balls (brand) | Movie  *(Alvin and the Chipmunks)* | Experimental group 1 was exposed to a scene of the movie where the product was placed frequently.  Experimental group 2 was exposed to a scene of the movie where the product was placed moderately.  Comparison group was exposed to a scene of the movie were no product placement appeared. | Choice  Significant effects of high frequency product placement on brand choice (no effects in the case of moderate frequency).  No significant interaction effects between age or prior view of the movie and exposure. | Low | |  |
| Naderer  (2016)  *International Journal of Advertising*  Austria [25] | 145 | 7.99  (1.17)  [6-11]  45.4% | Fritos (brand) | Cartoons *(Designed for the experiment with Powtoon software)* | 2x2 factorial design between subjects design [placement frequency (low vs. high), plot connection (present vs. absent)]  Control group (no food placement) was used but its data was not reported in this article. | Choice  Significant effects of high plot connection on brand choice.  No significant effects of placement frequency. | Low | |  |
|  |  |  |  |  |  |  |  | |  |
| Naderer  (2017)  *International Journal of Advertising*  Austria [32] | 363 | 10.55  (1.97)  [6-15]  52.9% | M&M´s (brand) | Movie  *(The Smurfs)* | Experimental group 1 (CPI condition) was exposed to a scene of a movie where the product is placed frequently and interacting with the character (food consumed).  Experimental group 2 (static condition) was exposed to another scene of the same movie where the product is placed frequently but the food is not consumed.  Comparison group was exposed to a scene of the movie where the target product placement did not appear. | Choice  Significant effects of product placements on brand choice.  Significant higher effects for CPI placements than for static placements.  No moderating effects neither of age nor movie familiarity. | Middle | |  |
| Naderer  (2018)  *Appetite*  Austria [26] | 175 | 8.41  (1.16)  [6-11]  51.4% | Mandarin  Gummy | Cartoons *(Designed for the experiment with Powtoon software)* | Experimental group 1 was exposed to a scene of a created cartoon where a healthy food (mandarin) was mentioned and placed frequently and interacting with the character.  Experimental group 2 was exposed to a scene of a created cartoon where an unhealthy food (gummy) was mentioned and placed frequently and interacting with the character.  Comparison group was exposed to a scene of the created cartoon were no food placements appeared. | Choice  Significant effects of unhealthy and healthy food placements on unhealthy food choice.  Healthy food placements did not trigger healthy food choices, on the contrary, healthy food placements triggered unhealthy food choices. | Low | |  |
| Neyens  (2017)  *Appetite*  Belgium [53] | 940 | 9.8  (2.4)  [6-14]  53.5% | Kellogg’s Coco Pops (brand) | Advergame  (*Collecting Coco-Pops playing the Mission Jungle 2)* | Experimental group 1 was exposed to watch a TV ad.  Experimental group 2 was exposed to play an advergame.  Comparison group was not exposed to any advertisement (control). | Choice  No significant effects of advergames on brand choice. | Middle | |  |
| Pempek  (2009)  *Archives of Pediatrics & Adolescent Medicine*  USA [54] | 30 | 9.5  (0.9)  [9-10]  50% | Orange juice  Banana  Apple  Carrots  Soda  Potato chips  Chocolate cookie  Chocolate candy bar | Videogame^3^  *(Designed for the experiment: Pac Man game)* | Experimental group 1 was exposed to a healthy advergame (participants gained points when the character ate healthy food and lost points when the character ate unhealthy food).  Experimental group 2 was exposed to an unhealthy advergame (participants gained points when the character ate unhealthy food and lost points when the character ate healthy food).  Comparison group was exposed to the advergame after selecting the snack (control). | Choice  No significant effects neither of healthy nor unhealthy food advergames (compared to control condition) on food choice.  Significant effects of unhealthy food compared to healthy food condition. | High | |  |
| Rosado  (2011)  *Revista Portuguesa de Marketing*  Portugal [55] | 133 | NR  (NR)  [7-11]  NR | Nesquik´s Chocolate Powder (brand) | Advergames  *(kart race in which the player gets points by collecting Nesquik powder)* | Experimental group played the food advergame.  Comparison group did not play any videogame (control). | Choice  Significant effects of the food advergame on brand choice. | Middle | |  |
|  |  |  |  |  |  |  |  | |  |
| Royne  (2017)  *Health Marketing Quarterly*  USA [56] | 64 | NR  (NR)  [6-11]  42% | Milk  Juice  Cola | Cartoon  *(SpongeBob SquarePants)* | Experimental group 1,2 and 3 were exposed to an episode of SpongeBob SquarePants where milk, juice or cola, respectively, was digitally inserted.  Comparison group was exposed to an episode of SpongeBob SquarePants where no drink appeared. | Choice  No significant effects of any drink condition on drink choice. | Middle | |  |
| Toomey  (2013)  *Young*  *Consumers*  USA [57] | 75 | 9.8  (1.29)  [8-12]  51.7% | Coca Cola zero (brand) | Sitcom  *(Designed for the experiment)* | Experimental group was exposed to a video where the actors drank Coca cola zero.  Comparison group was exposed to a video where the actors drank a non-branded drink. | Choice  No significant effects on brand choice. | High | |  |
|  |  |  |  |  |  |  |  | |  |
| Uribe  (2015)  *Appetite*  Chile [34] | 483 | NR  (NR)  [9-15]  54.9% | McDonald´s hamburger (brand) | Movie | Experimental group 1 was exposed to a part of the movie where McDonald’s audiovisual placements appeared. There were 2 breaks that did not include McDonald´s ads.  Experimental group 2 was exposed to a part of the movie where no McDonald’s placements appeared. There were 2 breaks that include McDonald´s ads.  Experimental group 3 was exposed to a part of the movie where McDonald’s audiovisual placements appeared. There were 2 breaks that include McDonald´s ads.  Comparison group was exposed to a part of the movie where no McDonald’s placements appeared. There were 2 breaks that did not include McDonald´s ads. | Choice  Significant effects of product placements, commercial ads and the combination of both on food category choice and brand choice.  Greater effects for the youngest group (children aged 9). | Middle | |  |
| Verhellen  (2014)  *Journal of Consumer Policy*  Belgium [58] | 125 | 11.98  (0.43)  [11-14]  NR | Ola ice cream (brand) | Advergame  (get the character collects as many Ola popsicles as possible) | Experimental group 1 was exposed to a 30-s trailer of Ola website.  Experimental group 2 played the Ola advergame.  Experimental group 3 was exposed to a 30-s trailer of Ola website and later the group played the Ola advergame.  Experimental group 4 was exposed to a traditional TV ad of Ola.  Comparison group was not exposed to any form of advertising messages. | Choice  No significant effects of treatments on brand choice. | Middle | |  |
| Villegas-Navas  (2019)  *International Journal of Environmental Research and Public Health* | 124 | 9.24  (1.19)  [7-11]  51.6% | Bacon  Burrito  Cookies  Gummy bears  Hot dog  Nachos  Mayonnaise  Pizza  Grapes  Asparagus  Lettuce  Coconut  Pineapple  Corn  Toast  Water | Cartoons  *(Adventure Time, All Hail King Julien, Fanboy and Chumchum, George of the Jungle, Gravity Falls, Pokemon, Phineas and Ferb, SpongeBob, The Amazing World of Gumball, The Jungle Book and The Ninja Turtles)* | Experimental group: Exposed to 16 scenes that contained 16 food placements (previously chosen randomly from a content analysis database)  Comparison group: Exposed to 16 scenes without food placements | Choice  Significant interaction effects between type of foods (low nutritional value foods) and age (children under age 9) | Middle | |  |

Abbreviations: Not reported (NR)
