# Supplementary material for: The Effects of Foods Embedded in Entertainment Media on Children’s Food Choices and Food Intake: A Systematic Review and Meta-Analyses
Source: Nutrients. 2020 Mar 31;12(4):964. doi: 10.3390/nu12040964 (PMC7230193; doi:10.3390/nu12040964)
Supplement: Supplementary file 1 [file nutrients-12-00964-s001.zip › Table S4_qualitative assessment.docx]

Effects of foods placed in entertainment media on children´s food choices and intake: a systematic review and meta-analyses.

Risk of bias assessment

| Authors  (Year) | Selection bias | | Performance bias | Detection bias | Attrition bias | Reporting bias | Other bias | Overall punctuation |
| --- | --- | --- | --- | --- | --- | --- | --- | --- |
|  | Random sequence generation | Allocation concealment | Blinding of participants and personnel | Blinding of outcome assessment | Incomplete outcome data | Selective reporting | Any conflict of interests declared? |  |
| Auty (2004) | Yes (0)  Participants were randomly assigned by their teachers to groups alphabetically by last name. | Unclear (1) | Unclear (1)  The researchers were careful to look elsewhere while each child was making his/her choice | Unclear (1) | Complete outcome data (0) | No (0) | No (0) | 3 |
| Brown  (2017) | No (2)  Children were assigned to groups based on convenience (mostly by child availability) and were not randomized | Unclear (1) | Unclear (1) | Unclear (1) | Complete outcome data (0) | No (0) | Unclear (1)  Dr. Bulik is a grant recipient from Shire and a consultant for Ironshore | 6 |
| Charry  (2014) | Unclear (1)  Children were randomly assigned to either the unimodal (visual placement) or the bimodal (audio-visual placement) condition. | Unclear (1) | Unclear (1) | Unclear (1) | Complete outcome data (0) | No (0) | No (0) | 4 |
| Dias  (2011) | Unclear (1) | Unclear (1) | Unclear (1) | Unclear (1) | Complete outcome data (0) | Unclear (1) | No (0) | 5 |
| Esmaeilpour  (2017) | Unclear (1)  Randomization not described | Unclear (1) | Unclear (1) | Unclear (1) | Complete outcome data (0) | Unclear (1) | No (0) | 5 |
| Folkvord  (2013) | Yes (0)  The teacher assigned the children (in alphabetical order) to the experimenter. | Unclear (1) | Yes (0)  The experimenter then left the room. | Unclear (1) | Complete outcome data (0) | No (0) | No (0) | 2 |
| Folkvord  (2014) | Unclear (1) | Unclear (1) | Unclear (1) | Unclear (1) | Complete outcome data (0) | No (0) | No (0) | 4 |
| Folkvord  (2015) | Unclear (1) | Unclear (1) | Unclear (1) | Unclear (1) | Complete outcome data (0) | No (0) | No (0) | 4 |
| Folkvord  (2016a) | Unclear (1) | Unclear (1) | Unclear (1) | Unclear (1) | Complete outcome data (0) | No (0) | No (0) | 4 |
| Folkvord  (2016b) | Yes (0)  The experimenter collected one child at a time from the classroom (in alphabetical order). | Unclear (1) | Unclear (1) | Unclear (1) | Complete outcome data (0) | No (0) | No (0) | 3 |
| Folkvord  (2017) | Yes (0)  The experimenter collected one child at a time from the classroom listed in alphabetical name order | Unclear (1) | Yes (0)  The experimenter left the room | Unclear (1) | Complete outcome data (0) | No (0) | Unclear (1) | 3 |
| Harris  (2012) | Unclear (1)  Children participated alone in one of three randomly assigned conditions | Unclear (1) | Unclear (1) | Unclear (1) | Complete outcome data (0) | No (0) | No (0) | 4 |
| Hudson  (2013) | Unclear (1) | Unclear (1) | Unclear (1) | Unclear (1) | Complete outcome data (0) | No (0) | No (0) | 4 |
| Mallinckrodt  (2007) | Unclear (1) | Unclear (1) | Unclear (1) | Unclear (1) | Complete outcome data (0) | No (0) | No (0) | 4 |
| Matthes  (2015) | Unclear (1) | Unclear (1) | Yes (0)  We used three different research assistants in order to prevent demand effects | Unclear (1) | Complete outcome data (0) | No (0) | No (0) | 3 |
| Naderer  (2016) | Unclear (1)  They randomly watched one of four versions of the stimulus movie with one experimenter supervising the screening. | Unclear (1) | Yes (0)  After stimulus presentation, three children were led to separate interview rooms by three researchers, while the fourth researcher stayed in the screening room with the remaining children and engaged them in a ball game. | Unclear (1) | Complete outcome data (0) | No (0) | No (0)  No potential conflict of interest was reported by the authors | 3 |
| Naderer  (2017) | Unclear (1)  Children in groups of three to four were randomly assigned to one of the three stimulus conditions | Unclear (1) | Unclear (1) | Unclear (1) | Complete outcome data (0) | No (0) | No (0)  No potential conflict of interest was reported by the authors. | 4 |
| Naderer  (2018) | Unclear (1)  The children were randomly exposed to one of three versions of the stimulus cartoon, with one experimenter supervising the screening. | Unclear (1) | Yes (0)  As there were four experimenters, three children were simultaneously interviewed in separate interview rooms, while the experimenter supervising the screening stayed in the screening room with the remaining children entertaining them with a ball game | Unclear (1) | Complete outcome data (0) | No (0) | No (0) | 3 |
| Neyens  (2017) | Unclear (1) | Unclear (1) | Unclear (1) | Unclear (1) | Complete outcome data (0) | No (0) | No (0)  No potential conflict of interest was reported by the authors. | 4 |
| Pempek  (2009) | Unclear (1)  each child was randomly assigned to 1 of the following 3 conditions (30 children) | Unclear (1) | No (2)  Participants worked individually with an experimenter | Unclear (1) | Complete outcome data (0) | Yes (2) | No (0) | 7 |
| Rosado  (2011) | No (2) | Unclear (1) | Unclear (1) | Unclear (1) | Complete outcome data (0) | No (0) | No (0) | 5 |
| Royne  (2017) | Unclear (1) | Unclear (1) | Unclear (1) | Unclear (1) | Complete outcome data (0) | No (0) | No (0)  No potential conflict of interest was reported by the authors. | 4 |
| Toomey  (2013) | No (2)  not totally random: After pairs were established, one participant in each pair was randomly assigned to the treatment group | Unclear (1) | Unclear (1) | Unclear (1) | (2) Incomplete outcome data | No (0) | No (0) | 7 |
| Uribe  (2015) | Unclear (1) | Unclear (1) | Unclear (1) | Unclear (1) | Complete outcome data (0) | No (0) | No (0) | 4 |
| Verhellen  (2014) | Unclear (1) | Unclear (1) | Unclear (1) | Unclear (1) | Complete outcome data (0) | No (0) | No (0) | 4 |
| Villegas-Navas (2019) | Unclear (1) | Unclear (1) | Unclear (1) | Unclear (1) | Complete outcome data (0) | No (0) | No (0) | 4 |
